# Mental health first aid for Indigenous Australians: using Delphi consensus studies to develop guidelines for culturally appropriate responses to mental health problems

**DOI:** 10.1186/1471-244X-9-47

**Published:** 2009-08-03

**Authors:** Laura M Hart, Anthony F Jorm, Leonard G Kanowski, Claire M Kelly, Robyn L Langlands

**Affiliations:** 1 Orygen Youth Health Research Centre, Centre for Youth Mental Health, University of Melbourne, Parkville, Victoria, Australia; 2 School of Psychology, Victoria University of Wellington, Wellington, New Zealand

## Abstract

**Background:**

Ethnic minority groups are under-represented in mental health care services because of barriers such as poor mental health literacy. In 2007, the Mental Health First Aid (MHFA) program implemented a cultural adaptation of its first aid course to improve the capacity of Indigenous Australians to recognise and respond to mental health issues within their own communities. It became apparent that the content of this training would be improved by the development of best practice guidelines. This research aimed to develop culturally appropriate guidelines for providing first aid to an Australian Aboriginal or Torres Strait Islander person who is experiencing a mental health crisis or developing a mental illness.

**Methods:**

A panel of Australian Aboriginal people who are experts in Aboriginal mental health, participated in six independent Delphi studies investigating depression, psychosis, suicidal thoughts and behaviours, deliberate self-injury, trauma and loss, and cultural considerations. The panel varied in size across the studies, from 20-24 participants. Panellists were presented with statements about possible first aid actions via online questionnaires and were encouraged to suggest additional actions not covered by the survey content. Statements were accepted for inclusion in a guideline if they were endorsed by ≥ 90% of panellists as *essential *or *important*. Each study developed one guideline from the outcomes of three Delphi questionnaire rounds. At the end of the six Delphi studies, participants were asked to give feedback on the value of the project and their participation experience.

**Results:**

From a total of 1,016 statements shown to the panel of experts, 536 statements were endorsed (94 for depression, 151 for psychosis, 52 for suicidal thoughts and behaviours, 53 for deliberate self-injury, 155 for trauma and loss, and 31 for cultural considerations). The methodology and the guidelines themselves were found to be useful and appropriate by the panellists.

**Conclusion:**

Aboriginal mental health experts were able to reach consensus about culturally appropriate first aid for mental illness. The Delphi consensus method could be useful more generally for consulting Indigenous peoples about culturally appropriate best practice in mental health services.

## Background

Australia's first National Mental Health and Wellbeing Survey, carried out in 1997, found that mental illnesses were common, associated with a high level of disability, but often not treated [1]. There are many possible reasons for the low rate of health service use. These include practical barriers such as financial cost, waiting lists and regional isolation. There are also other barriers such as an inability to recognise mental illness, or harbouring attitudes that are unfavourable to mental health care or to mental health professionals. In fact, lack of recognition and negative attitudes to treatment pose a significant barrier to service use, as research has shown that only one in three Australians are able to correctly label symptoms of a mental illness, and a majority do not consider interventions endorsed by health professionals, to be 'helpful' [2].

In 2001 the Mental Health First Aid program (MHFA) was established in response to the need for public education about mental illness and its treatment. MHFA offers a 12-hour course that applies a first aid action-plan model to mental illness [3]. Mental health first aid is defined as the help provided to a person developing a mental health problem or in a mental health crisis. First aid is given until appropriate professional treatment is received or the crisis resolves [4]. Through public education about helpful interventions for mental illness, this course aims to increase the public's mental health literacy and encourage the uptake of evidence-based treatment. Evaluations of the program have found that it is effective in increasing mental health literacy, changing beliefs about treatment to be more like those of health professionals, decreasing social distance from people with mental illness, increasing confidence in providing help to someone with a mental illness, increasing the amount of help provided to others and improving the mental health of participants [5]. The MHFA program has now been independently adapted by organisations in Aotearoa/New Zealand, Canada, England, Finland, Hong Kong, Japan, Northern Ireland, Scotland, Singapore, Thailand, USA and Wales [4,6].

In order to improve the quality of the mental health first aid techniques being taught to the public, research has been carried out to develop guidelines on what constitutes best practice first aid. This involved using the Delphi method to develop expert consensus. Expert consensus studies have been used as a practical alternative to randomised or controlled trials, which are not considered feasible or ethical for evaluating specific first aid techniques [7]. To date, guidelines have been developed for providing first aid in a range of mental health related crises, such as suicidal thoughts and behaviours [8], deliberate non-suicidal self-injury [9], and following traumatic events (Kelly, Jorm & Kitchener, *in submission*); as well as for a range of developing mental illnesses, such as psychosis [10], depression [11], eating disorders [12] and problem drinking (Kingston, Jorm, Lubman, Hart, Hides, Kelly, Kitchener & Morgan, *in submission*)

### The importance of culture

Research investigating the impact of culture on mental health has found that it is profound, multidimensional and complex [13]. Investigations of mental health care use have shown that ethnic minority groups are under-represented in psychiatric services, both within Australia and across other nations with developed health systems [14,15]. This trend occurs despite the prevalence of mental disorders in minority groups being either equal to, or greater than, those of the majority group. Research has also shown that the under-representation of minority groups in mental health care is not simply explained by inequalities in socioeconomic status across groups [15]. In fact, for cultural minorities other barriers, such as poor mental health literacy and culturally insensitive health services, play a much greater role in impeding use of services [16,17]. In recent years, many countries including the USA, Australia and Aotearoa/New Zealand have recognised the important role that culture plays in the identification, treatment and prevention of mental illness, and in response, have implemented standards of cultural competency in service delivery and specialised cultural adaptations of health education programs [18,19].

### Indigenous Australians

In Australia, the diverse groups of Aboriginal and Torres Strait Islander peoples, who constitute 2.3% of the population [20] exemplify the trend of poor mental health service use in the context of mental illness prevalence that is either equal to, or greater than, that of non-Indigenous Australians. Although prevalence estimates for mental illnesses in the Indigenous population are not well researched or documented (the two National Health and Wellbeing Surveys both elected not to collect specific information on the prevalence of mental disorders within the Australian Indigenous population [1,21]) there are some indirect measures which suggest that the mental health of Indigenous Australians is poor, recognition of mental illness within Aboriginal communities is low, and the prevalence of common mental illnesses such as depression and anxiety is high [22-30]. A recent health survey of Indigenous Australians reported that Aboriginal and Torres Strait Islander peoples are twice as likely as non-Indigenous Australians to report high or very high levels of psychological distress [31]. Furthermore, rates of suicide for the Indigenous population in Australia are estimated to be up to six times higher than rates for the non-Indigenous population [32].

While many programs aimed at improving mental health and reducing suicide in Indigenous Australians have been implemented in Aboriginal communities (e.g. Advanced Suicide Intervention Skills Training [33]), none have used an integrated approach to mental illnesses and associated crisis situations in a way that encourages early intervention and increased mental health literacy. The success of the MHFA course in increasing help-seeking behaviours, and the desperate need to provide Indigenous Australians with culturally appropriate training and education for improving mental health [34], led the Office of Aboriginal and Torres Strait Islander Health, a branch of the Australian Federal Government's Department of Health and Ageing, to fund the development of a cultural adaptation of the MHFA training program, specifically for Aboriginal and Torres Strait Islander Australians [35]. In 2007, an Aboriginal and Torres Strait Islander Mental Health First Aid training program (AMHFA) began teaching Australian Indigenous people a culturally adapted 14-hour course within Indigenous communities. The AMHFA course differs from the general MHFA course in recognising the historical, cultural and political forces that have affected Aboriginal mental health [35]. For instance, the course discusses how Australian Aboriginal people have endured centuries of racism, dispossession, violence, trauma and loss. The forced removal of communities from their land, the systematic denial of culture and language, the suppression of political and human rights, and the forced removal of children, have all contributed to an environment of poor mental health compounded by ongoing social and economic disadvantage [34]. In addition, the program acknowledges that Aboriginal people understand mental health within a unique cultural framework that is not necessarily complementary to the biopsychosocial model of western medicine [28,34]. Furthermore, it recognises that this cultural divergence can at times complicate the use of mental health services because Aboriginal people are either unable to find services that provide culturally sensitive treatment approaches, or fear accessing mental health services because historically these services have failed to provide care which incorporates and respects an individual's cultural world-view [28].

### The need for culturally specific mental health first aid guidelines

Although the existing Aboriginal Mental Health First Aid training program was developed through extensive consultation with Aboriginal people, it became apparent that the content of training would be improved by the development of best practice guidelines. In parallel to those developed for English-speaking [8-11] and Asian [6] countries, this research aimed to develop culturally appropriate guidelines for providing first aid to an Australian Aboriginal or Torres Strait Islander person who is experiencing a mental health crisis or developing a mental illness. By engaging Indigenous experts who work in the field of mental health, the research focused on the importance of culture and Indigenous experiences of mental illness.

## Methods

### The Delphi Method

Originally developed for technological forecasting, the Delphi technique has been used extensively within the last decade in health and social research, to enhance decision-making processes [7]. The Delphi method provides expert consensus on what constitutes best practice in scenarios that cannot be feasibly or ethically subject to a randomised controlled trial. The process involves questionnaires being sent out to a group of experts, who do not have to attend group meetings and can respond anonymously. Traditionally, the Delphi method has involved a number of iterations before consensus is achieved. Feedback is given at each stage in order to help experts assess their opinions against those of the group.

Development of these guidelines using the Delphi method involved four steps: (1) formation of the expert panel, (2) questionnaire development, (3) data collection and analysis, and (4) guidelines development. Five independent Delphi studies were conducted in order to produce mental health first aid guidelines on the following mental illnesses and crises:

▪ Depression

▪ Psychosis

▪ Suicidal Thoughts and Behaviours

▪ Deliberate Self-injury

▪ Trauma and Loss

A sixth guideline about the importance of understanding and respecting Aboriginal culture while providing mental health first aid, entitled Cultural Considerations and Communication Techniques (hereafter referred to as Cultural Considerations) was also developed to accompany the series.

#### 1. Panel formation

The research involved the recruitment of a panel of experts in the field of Aboriginal mental health. Participants were required to meet three inclusion criteria: to identify as an Aboriginal or Torres Strait Islander person; to be currently working in the field of mental health or to have had previous experience in the field; and to have an excellent knowledge of Aboriginal mental health and the types of issues involved when Aboriginal people seek assistance for their mental health problems. Potential candidates were considered to have sufficient expertise if they had authored materials on Aboriginal mental health (for instance teaching notes, journal articles, books or information leaflets), had attended and presented at meetings, conferences or training in Aboriginal mental health, or were known as respected professionals through networks with MHFA staff. Potential candidates were invited to participate via a face-to-face meeting, telephone call or email, and were sent a Participant Information Sheet prior to participation. Informed consent was implied by responding to online questionnaires. This research was granted human research ethics committee approval by the University of Melbourne. Participants were paid A$75 for each survey round completed.

#### 2. Questionnaire development

A systematic search was carried out on websites, online forums, information brochures, leaflets or hand-outs from service providers or information centres, medical journals and online databases for any written information about how to assist an Aboriginal person developing a mental disorder or experiencing a mental health crisis. There were three major sources of information. The first was a web-based search, which involved entering *a priori *key terms into an online search engine  and following the links to the first 50 sites listed. Additional File 1 displays a full list of search terms. Any links appearing on these websites, which the authors thought may contain useful information, were followed. The second was an academic journal database search (including Medline and PsycInfo), which presented relevant clinical research and literature reviews pertaining to the topic of interest (e.g [36,37]). The third was the National Libraries Australia database, which was used to identify key print texts located within Australian libraries (e.g. [38]). Recommendations from relevant mental health web sites (such as Australian Indigenous *HealthInfoNet*) were also searched.

Development of the first round questionnaires involved dividing the recommendations gleaned from the systematic search into sections based on common themes and developing statements that described first aid actions. For example, in the fact sheet called "Aboriginal Suicide Prevention Information", it states that "Expressing thoughts about death through drawings, stories or songs" may be a warning sign that the person is thinking about suicide. This sentence was included in the Round 1 Delphi survey on Suicide as the first aider action statement: *The first aider should be aware that expressions of thoughts about death through drawings, stories or songs, can be a warning sign that the person is thinking about suicide*.

In addition to these new items, statements that were presented to non-Aboriginal mental health experts in the previously conducted international Delphi studies were also incorporated into the questionnaires. The development of these non-Indigenous studies is described in detail elsewhere [8-11], so will not be elaborated on here, except to say that the recommendations for the general population were presented to the Aboriginal expert panel for consideration to ensure that any gaps in the Aboriginal-specific literature where still considered by the panel. This process was not followed for the Cultural Considerations guidelines as there was no precedent questionnaire.

The process of drafting statements and incorporating previous questionnaires involved a working group consisting of the authors, experts in the Delphi method and experts in Aboriginal mental health. When drafting first aid action statements from the literature, the working group attempted to remain as faithful as possible to the original wording of the information. Statements were only modified to ensure consistency of format, or where there was concern about the comprehensibility or cultural insensitivity of the information. Several draft questionnaires were produced before the group agreed on the statements which formed the final questionnaire for the first round. In total, three rounds of questionnaires were developed for each of six different topics (a total of 18 questionnaires).

#### 3. Data collection and analysis

Once panel members had been recruited, they were sent an electronic link to an online questionnaire, hosted by . Participants who were unable to complete the survey online were sent paper copies via mail. Allowing participants to choose between the two methods of delivery ensured that experts who had limited access to the internet were still able to participate in this research. Participants responded by rating how important the first aid action statements were to the development of a set of guidelines on providing culturally appropriate mental health first aid to an Aboriginal person. The questionnaire involved a five point scale including the following options:*Essential, Important, Don't know/depends, Unimportant *and *Should not be included*. In Round 1, panel members were also invited to make comments on any ambiguity or wording of the statements presented, and to suggest any new ones that had not yet been considered.

Once all participants had lodged their ratings, statements were placed into one of three categories.

• If between 90-100% of panel members rated a statement as either *Essential *or *Important*, the statement was endorsed as a guideline.

• If between 80-89% of panel members rated a statement as either *Essential *or *Important*, then the statement was entered into a second questionnaire to be re-rated.

• If neither of the above conditions were met, then the statement was excluded from the guidelines.

The protocol of using the *Essential *and *Important *ratings for categorisation was designed to allow selection of only those statements that were clear and broadly applicable for inclusion in the guidelines. Statements that were either unimportant to the provision of first aid, or dependent upon a situation or individual circumstance, were excluded because they did not constitute general principles that could instruct members of the public in how to provide first aid to an Aboriginal person.

Comments that were submitted by panel members were also analysed for any content that had not yet been addressed. To ensure comprehensibility and consistency, any additional ideas gleaned from these comments were written into first aid action statements and presented to the working group. Any statement that was judged by the group to be an original idea was included as a new statement in the second round questionnaire.

Once categorisation was complete, panel members were sent a report, which outlined the results of the questionnaire. The report consisted of a list of statements that had been endorsed, a list of statements that had been rejected, and a list of statements that were to be re-rated in the next questionnaire round. The statements to be re-rated were displayed with the group percentages for each possible rating, and also with the panel member's individual rating, so that panel members could compare their response to that of the group. Presentation of the report in this way allowed the panel members to decide whether to maintain or modify their ratings in the second round questionnaire.

**Figure 1:**
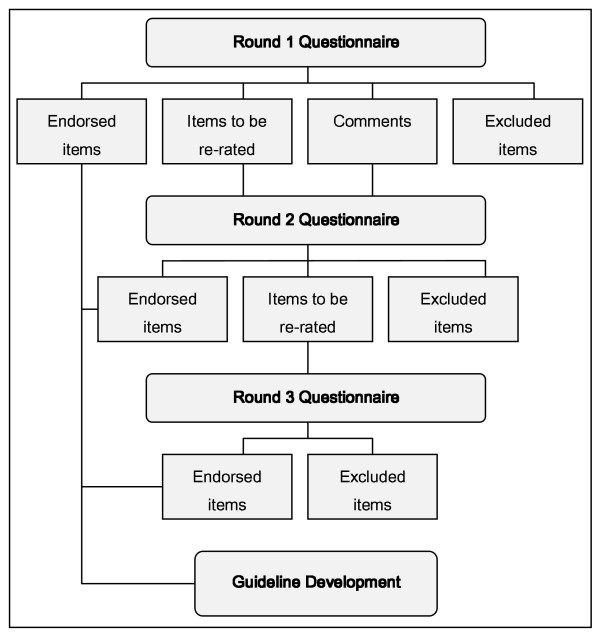
**The Delphi Method**. Figure 1 outlines how the Delphi method proceeded through three rounds of consultation with expert panel members in order to achieve consensus. If between 90-100% of panel members rated a statement as either *Essential *or *Important*, the statement was endorsed as a guideline. If between 80-89% of panel members rated a statement as either *Essential *or *Important*, the statement was then entered into a second round questionnaire to be rated again. To perform this next round of re-rating, participants were given a report outlining the results of the previous round, which showed their own individual ratings as well as the pooled ratings of the group. If the statement failed to meet the 90% endorsement level in the second re-rating round, it was then rejected. If a statement received a rating of 79% or less, it was rejected outright. Participants provided feedback in Round 1 which contributed to the edition of new items in Round 2. Only those items that fell into the re-rate category at the end of Round 2 were entered into a third a final round.

The same criteria for endorsing, excluding and re-rating statements were applied to the data collected in Round 2, with one exception. If a statement was re-rated in the second round and again failed to achieve a consensus of between 90 and 100 percent across the panel, it was then excluded. Only those statements that had been entered as new statements in Round 2, and afterward fell into the Re-rate category, were entered into a third round questionnaire. Figure 1 outlines how the Delphi method proceeded through three rounds of consultation with expert panel members in order to achieve consensus.

#### 4. Guidelines development

All statements endorsed as either *Essential *or *Important *by ≥ 90% of panel members were written into a guideline document. One author (LMH) drafted the guidelines by writing the list of endorsed statements into sections of prose based on common themes. Where possible, statements were combined and repetition deleted to reduce length. The draft was then presented to the working group, who edited the document to create a set of guidelines that were written in plain English and were easy to follow. A number of drafting iterations were completed before the group agreed upon a final document, a copy of which was sent to each panel member for review. Upon final endorsement by the expert panel, each guideline was printed in hard-copy A4 format for free distribution to interested panel members, Aboriginal Mental Health First Aid instructors, members of the public and attendees at health forums and conferences. The guidelines are also available for free download from the MHFA website: 

### Feedback from panel members

To the authors' knowledge, this research is only the second time the Delphi method has been used to develop expert consensus among Aboriginal people in Australia [39]. To assess the panel members' satisfaction with the research method, participants were invited to complete an online feedback survey at the end of the six Delphi studies. Respondents were encouraged to comment on the appropriateness of the contact methods, research methods, language and concepts used throughout the studies. They were also asked how culturally appropriate and useful they thought the developed guidelines would be to Aboriginal people in the future.

The feedback survey contained 48 statements that described the research experience (e.g. *I thought the use of email and internet was a good medium for data collection*). Participants were asked to respond by selecting where their opinion fell on a 5-point scale of agreement; *Strongly Agree, Agree, Neither Agree nor Disagree, Disagree*, and *Strongly Disagree*.

## Results

### Expert panel members

31 panel members were recruited and a total of 28 participants (15 female, 13 male, age range = 31 to 61 years) completed at least three questionnaires. Table 1 outlines how many panel members responded to each round of each topic. There was a high retention rate both across rounds of questionnaires and across topics. The majority of panel members responded to more than 9 rounds; 18 participants (64.3%) completed 10-18 questionnaires.

**Table 1:** Number of respondents for each round of each questionnaire topic

	Depression	Psychosis	Cultural considerations	Suicidal thoughts & behaviours	Deliberate self-injury	Trauma & loss
Round 1	20	20	24	24	24	24
Round 2	18	18	20	20	20	21
Round 3	17	17	19	19	19	21

Participants were recruited from across Australia including: the Australian Capital Territory (n = 2), New South Wales (n = 11), the Northern Territory (n = 1), Queensland (n = 7), South Australia (n = 1), Victoria (n = 4) and Western Australia (n = 2). Tasmania was the only state without representation on the panel. Having a geographical spread of panel members was thought to be important for the representation of different experiences and attitudes of Aboriginal communities across Australia. It is therefore important to note that no participants identified as Torres Strait Islander. According to the 2006 Australian Census, 90% of the Australians who identified as Indigenous were of Aboriginal origin only, 6% were of Torres Strait Islander origin only and 4% were of both Aboriginal and Torres Strait Islander origin [20]. Given that Torres Strait Islander people constitute such a small percentage of the Indigenous population, it is not surprising we found it difficult to recruit a representative from this community.

Participants were employed in a range of different services across the mental health field, including: private psychology clinics, Aboriginal medical services, government health services, universities, cultural resource and counseling services, prisons, social services, and drug and alcohol services.

The panel was very experienced, with more than half having between 6-10 years experience in the mental health field (5 years or less = 12.5%, 6-10 years = 62.5%, 11-15 years = 12.5%, 16-20 years = 12.5%). In addition, approximately one quarter of panel members had obtained a post-graduate degree (Certificate level = 11.6%, Diploma = 11.76%, Bachelor Degree = 47.06%, Bachelor degree with honours = 5.88% Graduate Diploma = 5.88%, Masters degree 17.65%, PhD = 0.00%).

### Endorsed statements

Of the 1,016 statements that were presented to panel members across the 6 Delphi studies, 536 were endorsed as either *Essential *or *Important *to the development of guidelines on providing mental health first aid to an Aboriginal person. A list of all endorsed statements can be found in Additional File 2, Tables S1 - S6. The number of statements presented in each first round questionnaire differed markedly across topics and was largely determined by the amount of accessible literature on the area. There is, for instance, a large and culturally relevant literature on the issue of suicidal thoughts and behaviours, however, there is a notable dearth of culturally relevant information on issues such as depression and deliberate self-injury. The largest questionnaires were those on trauma and loss, and psychosis. Table 2 lists the number of statements presented in each Delphi study.

**Table 2:** Number of statements presented, endorsed and rejected in each Delphi study

		Depression	Psychosis	Cultural considera- tions	Suicidal thoughts & behaviours	Deliberate self-injury	Trauma & loss
Round	New statements	155	143	42	125	76	211
1	Statements being re-rated	0	0	0	0	0	0
	Total number of statements	155	143	42	125	76	211
	Statements Endorsed	72	125	24	30	16	125

Round	New statements	13	10	27	20	22	18
2	Statements being re-rated	26	32	8	17	12	32
	Total number of statements	39	42	35	37	34	50
	Statements Endorsed	21	24	25	22	13	27

Round	New statements	0	0	0	0	0	0
3	Statements being re-rated	3	2	5	4	4	9
	Total number of statements	3	2	5	4	4	9
	Statements Endorsed	1	2	3	1	2	3

Total number of statements		197	187	82	166	114	270
**Total number of endorsed statements**		**94**	**151**	**52**	**53**	**31**	**155**
Total number of rejected statements		103	36	30	113	83	115

### Rejected statements

Some statements were strongly rejected by the panel, with a majority of participants rating a statement as either *Unimportant *or *Should not be included *(see Additional File 2, Table S7, for a list of strongly rejected statements). Across the 6 Delphi studies 36 items were rejected with strong consensus (50% or more of panel members rated an item as either *Unimportant *or *Should not be included*). The majority of strongly rejected items came from the suicide and deliberate self-injury surveys, while the psychosis, cultural considerations and trauma and loss surveys received no strong rejections.

Other statements were rejected because there was disagreement within the panel. For instance, some statements failed to be endorsed because, even after a second rating, the statement just failed to achieve 90% consensus.

### Panel member feedback

Eighteen of a possible 24 participants responded to the feedback survey. The percentage of panel members responding in each category are shown in Table 3. Of particular interest were the responses to statements that were designed to assess the cultural appropriateness, the utility and perceived quality of the guidelines produced. For instance, in response to the statement *I would recommend the guidelines to other people*, all participants responded with either *Strongly Agree *or *Agree*. In addition, 88.9% of the panel responded with either *Strongly Agree *or *Agree *in response to both statements *I thought the guidelines were culturally appropriate *and *I believe the guidelines will benefit Aboriginal people*. While these results are promising, they are not unexpected, given that the panel members were involved in the development of the guidelines. The results would be well supported by further evaluation of the utility of the guidelines in Indigenous communities.

**Table 3:** Selected statements from the panel member feedback survey

Feedback statement	Strongly Agree	Agree	Neither Agree nor Disagree	Disagree	Strongly Disagree
I thought the Guidelines were easy to follow.	44.4	44.4	5.6	0.0	0.0
I thought the Guidelines were too long.	0.0	5.6	22.2	61.1	11.1
I thought the guidelines used appropriate language.	22.2	55.6	22.2	0.0	0.0
I thought the language used in the guidelines was too clinical.	0.0	5.6	27.8	55.6	11.1
I thought the guidelines covered the appropriate issues.	33.3	61.1	5.6	0.0	0.0
I thought the guidelines were culturally appropriate.	27.8	61.1	11.1	0.0	0.0
I believe the guidelines will benefit Aboriginal people.	55.6	33.3	5.6	0.0	0.0
I would recommend the guidelines to other people.	55.6	44.4	0.0	0.0	0.0
I thought the time commitment was appropriate.	16.7	66.7	0.0	16.7	0.0
I thought the remuneration was appropriate.	27.8	61.1	11.1	0.0	0.0
I thought participating in this research was worthwhile.	83.3	16.7	0.0	0.0	0.0
I enjoyed participating in the Delphi research.	61.1	33.3	5.6	0.0	0.0
I believe the Delphi process can be of benefit to Aboriginal people.	44.4	38.9	11.1	5.6	0.0
I would recommend the Delphi method for other research projects for Aboriginal people.	44.4	38.9	11.1	5.6	0.0

Statements regarding the appropriateness of the Delphi research method also received a high level of agreement, with 83.3% of participants responding with either *Strongly Agree *or *Agree *to the statements *I believe the Delphi process can be of benefit to Aboriginal people *and *I would recommend the Delphi method for other research projects for Aboriginal people*.

## Discussion

By engaging Aboriginal people who are experts in the field of mental health, this research aimed to develop culturally appropriate guidelines for providing mental health first aid to an Australian Aboriginal or Torres Strait Islander person. Despite diverse backgrounds, the expert panel was able to reach consensus on a range of first aid techniques, from offering a cup of tea or coffee to a person who has experienced trauma, through to talking about the sensitive issue of suicide.

Across the different Delphi topics, statements were rejected and endorsed at different rates. For instance, the psychosis study proposed a total of 187 first aider action statements, of which 81% were endorsed and 19% were rejected. In contrast, the deliberate self-injury study proposed a total of 114 statements, of which 27% were endorsed and 73% were rejected. While it might be expected that the Delphi studies containing the most cultural information may have had the highest rates of endorsement (e.g. trauma and loss or cultural considerations), in fact the pattern of endorsement mirrored that of other international Delphi studies, which have found differences in the strength of established expert consensus [8-11]. That is, because of wide-ranging and effective research into first episode psychosis, there is a strong consensus on how emerging psychosis should be managed. Conversely, due to a dearth in controlled trials and treatment studies, there is little expert consensus on how deliberate self-injury should be treated in a clinical context, let alone managed in the community.

Although each Delphi study addressed a different mental health issue, there were two themes that appeared in each survey and subsequent guidelines. The first theme was about how the person providing first aid needed to understand and assess symptoms of mental illness within the cultural context of the person they were helping. The second was the essential role that family and community play in promoting and protecting the health and wellbeing of individuals with mental health problems.

### Understanding symptoms of mental illness within a cultural context

A particular concern of culturally appropriate mental health first aid is to check, or to understand, the cultural norms of the community, before assuming that an Indigenous person is displaying symptoms of mental illness. All of the Delphi study surveys included statements about symptom recognition in the context of culture. For instance, the depression study included the statement - *The first aider should take into consideration the spiritual and/or cultural context of the person's behaviours*. The psychosis study included the statement - *The first aider should be aware of what constitutes culturally appropriate behaviours so that they don't misinterpret such behaviours as symptoms of psychosis (e.g. in some communities, limited eye contact is expected behaviour)*. The suicidal thoughts and behaviours study included the statement - *The first aider should learn about the behaviours that are considered warning signs for suicide in the person's community*. And the deliberate self-injury study included the statement - *The first aider should be aware that pathological self-injury, such as cutting and burning, is fundamentally different to ritualistic, culturally accepted Aboriginal ceremonial or grieving practice*.

Each of these statements were endorsed by panel members and incorporated into the guideline documents. That each of the developed guidelines contain information about the need for the person providing first aid to be mindful of cultural norms, before assuming that someone is experiencing a mental health problem, suggests that this is a cornerstone of culturally appropriate first aid. Importantly, from the feedback provided by panel members during the Delphi studies, this principle was not only seen as important for non-Indigenous people providing first aid to Indigenous people, it was also seen as crucial for any Indigenous person who was assisting outside their own community.

While panel members endorsed this idea of culturally appropriate first aid, they did so with a caveat. Panel members comments suggested that individuals providing first aid need not get so immersed in the need for cultural awareness that they lose sight of the physical and emotional needs of the person they are assisting; as one panel member's comments suggest: *"Whilst having attested to the importance of these cultural awareness items, they sum to a maxim that it's crucial to take time to become familiar with local beliefs and norms, [yet] it is actually counter-productive to think one has to be an anthropologist - across the minutiae of all Australian Indigenous cultures..."*. This idea was also especially apparent in the study on trauma and loss. In the Round 1 and 2 surveys, panel members were presented statements about seeking culturally appropriate professional help. Allowing an Aboriginal person to seek out a professional who is trained or experienced in treating Aboriginal people and their experiences of trauma, was seen as particularly important in facilitating recovery. As the Delphi progressed, however, it became apparent that any rigid statements about the need for culturally appropriate professional help were not going to be endorsed, because they alone precluded the right of the person receiving care to seek help that is close to their home and community, and which suits their individual needs. The Trauma and Loss guidelines therefore not only include the statement *Suggest that the person see a professional who is trained or has experience in working with Aboriginal people and their experiences of trauma and loss*, it also contains the additional caveats: *It is important to note that counselling suitable for Aboriginal people may be quite difficult to find or gain access to, as there is a shortage of appropriately trained Aboriginal psychologists and counsellors. If this is the case, you can engage other options; *and *Most importantly, encourage the person to find someone who will help them tell their story and who the person can trust and feel comfortable talking to*. So while this research supports the importance of providing culturally appropriate first aid, it also asserts that when assisting an Indigenous person with a mental health problem, it is equally important to meet their individual needs, regardless of their cultural identity.

### The role of family and community

The statements endorsed by the panel and the feedback comments submitted in the first round of each study revealed that, to provide culturally appropriate first aid, the person assisting should facilitate additional support for the person in their care, by encouraging positive relationships with family and community members, while upholding the person's right to confidentiality. For example, the Psychosis guideline contains the statement - *Encourage the person to take a support person, such as a family member, to their appointment. If you wish to help the person contact their family, be aware that you must ask the person if its okay for you to talk to family*. The Cultural Considerations guideline contains the statement - *Try to get the person's family involved in supporting them until they get better, but in doing so, you must uphold the person's right to confidentiality*. The Trauma and Loss guideline contains the statement - *Whether or not the person seeks professional help, you should encourage them to identify sources of support. These may include community members, support groups and men's or women's groups*.

While facilitating support for a person experiencing a mental health problem is recommended as a first aid action in the guidelines produced by previous Delphi studies for English-speaking countries (for instance see *Depression: First Aid Guidelines*) [31], the focus of previous guidelines has been on developing a supportive relationship between the person providing the first aid and the person receiving care. The Delphi studies conducted in this research, reveal that it is important to establish an additional support person, who can act as a mentor or carer.

This additional first aid strategy appears to have arisen for a number of important reasons. For example, many Aboriginal people live in regional or remote communities with limited access to mental health care. Establishing a positive and trusting relationship with another person is a way to establish additional psychological support that may otherwise be unavailable. This is demonstrated by the Cultural Considerations guidelines, which state: *Establishing a network of support for an Aboriginal person is a very important step in helping them resolve their mental health crisis, especially if access to professional support or mental health services is limited*. Another reason for including the facilitation of additional support is to protect against further psychological distress by enhancing the person's social and emotional wellbeing. This is exemplified in the statement: *Discuss with the person what their interests and activities are and encourage participation in any group activities that will help them to develop feelings of purpose, belonging and achievement*, which also appears in the Cultural Considerations guidelines.

### Acceptance of research outcomes and plans for dissemination

The feedback from panel members demonstrated that support for the Delphi method and the guidelines it produced was very strong (see Additional File 2 Table S4). However, for the developed guidelines to be successful, they need to have a direct impact on the Indigenous communities within Australia. The culturally specific 14-hour course developed by the Aboriginal Mental Health First Aid program is one avenue to achieve this impact.

Since its inception in 2007, the AMHFA course has been presented to over 1,936 Australians. When the teaching materials are revised to reflect the consensus on first aid techniques developed by these Delphi studies, the information in these guidelines will reach a significant number of Indigenous Australians. Furthermore, the detailed material presented in the guidelines will be organised under the MHFA ALGEE action plan [40] and will have associated teaching activities such as role plays and DVD clips. A pictorial flip-chart is also planned. The dissemination of the guideline information in this way reduces the need for English literacy and thus makes the first aid information more accessible to Indigenous people who may only use English as a second (third or fourth) language.

In addition to the AMHFA course, the *beyondblue: the national depression initiative *has developed a national dissemination program whereby copies of the guidelines will be sent free of charge to health, education and community resource centres across Australia, who engage Indigenous clients. Furthermore, the guidelines will be made available to order free from *beyondblue*. Given that a number of important stake-holders in Aboriginal mental health were involved in the guideline research as expert panel members, and that the feedback survey demonstrated these stake-holders approve of the research outcome and are willing to recommend the guidelines to others, the authors believe the national dissemination project will be successful in presenting the guidelines to a large number of people who care for Indigenous Australians with mental health problems.

One limitation of the current research is the lack of pertinent information for members of the community who wish to provide mental health first aid to young Indigenous Australians. Given that, in 2001, 39% of Indigenous people were under 15 years of age, compared with 20% of non-Indigenous people, providing first aid resources for young Aboriginal and Torres Strait Islander Australians is an important task for AMHFA. It was however, a considered decision of the research team to develop guidelines focused on adults. Now that the validity and acceptability of the research method and outcomes have been established, it is hoped that future Delphi studies will be able to develop best practice guidelines for providing assistance to young Indigenous people developing a mental illness or experiencing a mental health crisis.

Despite provisional support from the experts involved in the guideline production, and a highly structured dissemination plan, only further evaluation of first aid outcome will elucidate whether or not the information developed by this research is effective in decreasing the barriers to mental health care faced by many ethnic minority groups in Australia, such as poor mental health literacy, and ultimately increasing the use of health services by Indigenous people.

## Conclusion

Aboriginal mental health experts were able to reach consensus about what are appropriate first aid actions for a range of mental illnesses and mental health crisis situations. The Delphi research method was able to develop a resource, which describes for the first time, what constitutes culturally appropriate best practice first aid for Aboriginal and Torres Strait islander people with mental health problems. Through a range of dissemination programs, the information in the guidelines will be made available to Indigenous and non-Indigenous people throughout Australia.

According to the outcomes of this research, to provide culturally appropriate first aid to an Australian Aboriginal or Torres Strait Islander person, individuals must be aware of relevant cultural factors in mental illness, such as cultural behaviours that may mimic symptoms of mental illness, the important role of family and community, and the need to facilitate supporting relationships. These findings are consistent with the broader literature on culturally appropriate care [17,18].

This research also demonstrates that the Delphi consensus method is a framework that can be used to guide the improvement of mental health services for Indigenous people, as it allows participants to engage in a culturally appropriate way, and produces resources that can of benefit to their community.

## Competing interests

LK is the Deputy Director of the Australian Mental Health First Aid Training and Research program, and Manager of the Aboriginal Mental Health First Aid (AMHFA) training program. AFJ is the scientific director of the Australian Mental Health First Aid training and research program. CMK is the Manager of the Youth Mental Health First Aid training program. The publication of this manuscript may benefit the AMHFA training program by advertising the concept of mental health first aid for Aboriginal Australians.

## Authors' contributions

LMH carried out the systematic literature search, was involved in panel member recruitment, drafted the surveys, carried out the data collection and analysis, chaired the working group which discussed and modified the survey and guideline drafts, drafted the guidelines, and drafted the manuscript. AFJ participated in the conception and design of the Delphi research protocol, was co-ordinator of the research, participated in the working group and helped with the drafting of the manuscript. LGK was involved in design and co-ordination of the study, participated in panel member recruitment and participated in the working group. CMK was involved in design and co-ordination of the study, participated in panel member recruitment and participated in the working group. RLL participated in the systematic literature search, panel member recruitment, drafting the surveys, and data collection and analysis. All authors read and approved the final manuscript.

## Pre-publication history

The pre-publication history for this paper can be accessed here:



## Supplementary Material

Additional file 1 **Search terms**. A list of the key search terms used in the systematic literature search for all six Delphi studies. Click here for file

Additional file 2 **Statement lists**. A list of endorsed statements, and a list of strongly rejected statements, from all six Delphi studies shown in a series of tables. Click here for file
