# Supplementary material for: Mental health first aid for Indigenous Australians: using Delphi consensus studies to develop guidelines for culturally appropriate responses to mental health problems
Source: BMC Psychiatry. 2009 Aug 3;9:47. doi: 10.1186/1471-244X-9-47 (PMC2729076; doi:10.1186/1471-244X-9-47)
Supplement: Additional file 1 — Search terms. A list of the key search terms used in the systematic literature search for all six Delphi studies. [file 1471-244X-9-47-S1.pdf]

Additional File 1

Table 1 - Key Search Terms

Depression	Psychosis	Suicidal thoughts & behaviours	Deliberate self-injury	Trauma and loss
Aboriginal<br>Indigenous	Aboriginal<br>Indigenous	Aboriginal<br>Indigenous	Aboriginal<br>Indigenous	Aboriginal<br>Indigenous
Early Intervention<br>Help<br>First Aid	Early Intervention<br>Help<br>First Aid	Early Intervention<br>Help<br>First Aid	Early Intervention<br>Help<br>First Aid	Early Intervention<br>Help<br>First Aid
Depression<br>clinical<br>depression<br>major depressive<br>disorder<br>depression<br>carers<br>support<br>depression<br>sufferers	Psychosis<br>first-episode psychosis<br>psychosis family friends<br>psychosis carer<br>Schizophrenia<br>Schizophrenia carer<br>hearing voices, hallucinations,<br>delusions<br>psychosis violence<br>what to do psychosis violence	Suicide, suicidal,<br>suicidality<br>help for suicide<br>suicide intervention<br>suicide family<br>friends<br>suicidal ideation<br>suicide behaviour	Self harm, self injury<br>non-suicidal self<br>injury<br>deliberate self harm,<br>deliberate self injury<br>parasuicide<br>cutting	Trauma<br>trauma excluding wounds or<br>injuries<br>post-traumatic stress disorder<br>PTSD<br>stress disorders
