# Supplementary material for: Mental health first aid for Indigenous Australians: using Delphi consensus studies to develop guidelines for culturally appropriate responses to mental health problems
Source: BMC Psychiatry. 2009 Aug 3;9:47. doi: 10.1186/1471-244X-9-47 (PMC2729076; doi:10.1186/1471-244X-9-47)
Supplement: Additional file 2 — Statement lists. A list of endorsed statements, and a list of strongly rejected statements, from all six Delphi studies shown in a series of tables. [file 1471-244X-9-47-S2.pdf]

Additional File 2

Table 1 - Endorsed statement from the Delphi study "First Aid for Depression"

Round endorsed	Endorsed Statements
1	The first aider should be able to recognise the symptoms of depression.
1	If the first aider notices changes in mood, the behaviour (e.g. drinking and/or drug abuse), energy level, habits (e.g. an increase or decrease in sleep or appetite) or the personality of someone they know, they should consider depression as a possible reason for these changes.
1	If the first aider knows someone who is showing signs of depression, they must not ignore the symptoms or assume that the depression will just go away.
1	The first aider should remain aware that each individual is different and not everyone who is experiencing depression will show the typical signs or symptoms of depression.
1	When helping someone who may be depressed, the first aider must not assume that the person is experiencing depression simply because s/he observes the symptoms of depression.
1	The first aider should not lie or make excuses for the depressed person's behaviour as this may delay getting assistance.
1	The first aider needs to learn about depression in order to help someone with depression.
1	The first aider should take time to find out information about depression such as its causes, its symptoms, how it can be treated, and what services are available in their local area.
1	The depressed person may not have the energy or cognitive strength to thoroughly investigate the resources available on their own. The first aider should help the depressed person find out information about depression.
1	If the first aider does give the person information about depression, they must ensure that the resources are accurate and appropriate to the person's situation.
1	The first aider should respect how the person interprets their symptoms.
1	The first aider should convey to the person that depression is very common.
1	If the person doesn't feel comfortable talking to the first aider, s/he should encourage them to discuss how they are feeling with someone else.
1	The first aider needs to let the depressed person know s/he is concerned about them and is willing to help.
1	If the first aider is worried about someone who may be depressed, s/he should give the person appropriate opportunities to talk and let the person choose the moment to open up.
1	The first aider should choose a suitable time when s/he and the depressed person are both available to talk, as well as a space where they both feel comfortable.
1	The first aider should ask the person about their mood.
1	If the person says that they are feeling sad or down, the first aider should ask them how long they have been feeling that way.
1	Everybody feels down or sad at times, but the first aider should be able to recognise when depression has become more than a temporary experience for someone and when to encourage that person to seek professional help.
1	The first aider should encourage the person they are helping to get professional help as early as possible.
1	The first aider should help the depressed person find a doctor, therapist or psychiatrist.
1	If the person does not know where to get help, the first aider should offer to help them seek assistance.

1 If the first aider accompanies the person to the doctor's appointment, they must not take over completely; a person with depression needs to make their own decisions as much as possible.

1 Depression is often not recognised by health professionals; it may take some time to get a diagnosis and find a healthcare provider with whom the depressed person is able to establish a good relationship. The first aider should encourage the person s/he is helping not to give up.

1 The first aider can help someone who may be depressed by encouraging them to get an appropriate professional diagnosis and effective treatment.

1 The first aider should ask the person if they need help to manage how they are feeling.

1 The first aider should ask the person if they have tried to get help.

1 The first aider should discuss the options that the person has for seeking help and should encourage them to use these options.

1 The first aider should tell the depressed person about the way treatment might help.

1 The first aider needs to recognise that the person’s ability and desire to use self-help strategies will depend on their interests and the severity of their depression.

1 The first aider should ask the person if they are interested in talking about self-help strategies. If the person says yes, the first aider could provide them with a range of information about self-help strategies.

1 The first aider should help the depressed person by knowing what self-help strategies are useful and then recommending these.

1 The first aider should not be too forceful when trying to encourage the depressed person to use self-help strategies.

1 The first aider needs to respect the person’s autonomy while considering the extent to which the person is able to make decisions for themselves, and whether the person is at risk of harming themselves or others.

1 Everyday activities like cleaning the house, paying bills, or feeding the dog may seem overwhelming to a depressed person. The first aider should acknowledge that a person with depression is not “faking”, “lazy”, “weak” or “selfish.”

1 The first aider should not be hostile or sarcastic when the depressed person attempts to be responsive; s/he should accept these responses as the best the person has to offer at that time.

1 The first aider should not adopt an over-involved or over-protective attitude towards someone who is depressed.

1 The first aider should try not to show the depressed person how they feel, if the person’s depression is bringing them down.

1 The depressed person genuinely needs additional love and understanding to help them through their illness so the first aider should be empathetic, compassionate and patient.

1 The first aider needs to realise that there's no point in just telling someone with depression to get better.

1 The first aider should keep in mind that the depressed person can't “snap out of it” or “get over it.”

1 The first aider should not be afraid to encourage the person to talk about their feelings, symptoms and what is going on in their mind.

1 The first aider should not tell the depressed person that they just need to stay busy or get out more.

1 The first aider should not trivialise the depressed person’s experiences by pressuring them to “put a smile on their face,” to “get their act together,” or to “lighten up”.

1 The first aider should not belittle or dismiss the person's feelings by attempting to say something positive like, "You don't seem that bad to me."

1 The first aider should let the depressed person know that they are not weak or a failure because they have depression, and that s/he doesn’t think less of

them as a person.

1 The first aider should remind the person that this is an illness and that they are not to blame for feeling “down.”

1 If the person is unrelentingly pessimistic, the first aider should try to point out the positive things that are happening.

1 When asking questions, the first aider should make them open-ended so the depressed person can say what they want to.

1 The first aider needs to be aware that silence may be better than talking. If the person doesn’t want to talk, the first aider could just be with them for a while.

1 The first aider should avoid speaking to the depressed person with a patronizing tone of voice and should not use overly-compassionate looks of concern.

1 The first aider should be honest with the depressed person.

1 The first aider should ask the person whether what they are doing is helpful, and what else they could do to help.

1 The first aider should let the person know that s/he is available to talk when they are ready, and should not put pressure on the person to talk right away.

1 The first aider should be an "active listener" and reflect back what the depressed person has said before responding with their own thoughts

1 The first aider should not nag the person to try to get them to do what they normally would.

1 The first aider needs to encourage the depressed person that, with time and treatment, they will feel better.

1 The first aider should respect the person’s privacy and confidentiality unless they are concerned that the person is at risk of harming themselves or others.

1 The first aider needs to be patient, persistent and encouraging when supporting someone with depression.

1 The first aider needs to let the person with depression know that they will not be abandoned.

1 The first aider needs to recognise that each person’s situation and needs are unique.

1 The first aider can help someone with depression by listening to them without expressing judgement.

1 The first aider needs to listen carefully to the depressed person even if what they tell her/him is obviously not true or is misguided.

1 Although the depressed person may not be communicating well, and is probably speaking slower and less clearly than usual, the first aider must be patient and must not interrupt.

1 If the depressed person is repetitive, the first aider should try not to get impatient but rather keep trying to be as supportive as possible.

1 A depressed person is often overwhelmed by irrational fears. The first aider needs to be gently understanding of someone in this state.

1 The first aider should resist the urge to try to cure the person’s depression or to come up with answers to their problems.

1 The first aider should let the person know in advance that they will intervene and seek professional help for the person if they ever believe the person’s life may be in danger.

1 The first aider should let the person know that if they change the mind in the future about seeking help, they can contact the first aider.

1 The first aider must respect the person’s right not to seek help at all times unless the first aider believes that they are at risk of harming themselves or others.

1 The first aider should only intervene without permission when the depressed person’s life is in danger.

1	The first aider should find out if there are specific reasons why the depressed person does not want to seek professional help (e.g. concerns about finances, not having a doctor they like, or being worried they will be sent to hospital) as sometimes such reasons are based on mistaken beliefs, or can be overcome with help.
2	The first aider should take into consideration the spiritual and/or cultural context of the person's behaviours.
2	The first aider should be aware of what constitutes culturally appropriate behaviours so that they don't misinterpret such behaviours as symptoms of depression (e.g. in some communities, limited eye contact is expected behaviour).
2	When considering giving the person information about depression, the first aider should be mindful of the severity of the person's symptoms.
2	The first aider should remain aware that some Aboriginal People cannot read well and may need assistance with pamphlets and books.
2	Contrary to myth, talking about depression makes things better, not worse. If the first aider thinks someone may be depressed and needs help, the first aider should say something to that person.
2	The first aider should ask the person if they are feeling depressed.
2	The first aider should be aware of Aboriginal Health Services and the roles of elders and/or healers in the community.
2	The first aider should encourage the person to first see a doctor for potential diagnosis and treatment.
2	The first aider must not push the person into seeking professional help before they are ready, unless there is a specific risk of harm to self or others.
2	If the person has sought professional help, the first aider should ask them if they understood what was said to them in order to clarify any medical jargon used.
2	The first aider should ask the person what they have done in the past to help themselves cope and whether they could use such strategies to help themselves now.
2	The first aider should give the person the phone numbers of support lines e.g. Lifeline.
2	The first aider should ask the person if they would like any practical assistance with tasks but should be careful not to take over or encourage dependency.
2	The first aider should offer the depressed person kindness and attention, even if it is not reciprocated.
2	The first aider should be consistent and predictable in their interactions with the depressed person.
2	The first aider needs to realise that depression is a medical illness and remember that the person cannot help being affected by depression.
2	The first aider should avoid using the words "I know how you feel" or "I understand"; it is unlikely that they really do comprehend the depths of the person's sadness unless they have also been diagnosed with depression.
2	The first aider should not tell a depressed person that they are unpleasant to be around, even if s/he feels that way.
2	It is more important for the first aider to be genuinely caring than for them to say all the "right things".
2	The first aider should be mindful not to use jargon and should keep their language simple and applicable to the local community.
2	Sometimes the person may need time to accept the need for treatment. The first aider should slowly and respectfully persist in trying to get the person to seek help.
3	The first aider should be aware of the different conceptualisations of depression and the terminology used to describe these concepts in Aboriginal communities.

Table 2 - Endorsed statements from the Delphi study "First Aid for Psychosis"

Round endorsed	Endorsed Statements
1	The first aider should be able to recognise the early warning signs and/or symptoms of psychosis.
1	The first aider should realise that although warning signs and/or symptoms of psychosis are often not very dramatic on their own, taken together they may suggest that something is not quite right.
1	The first aider should not ignore or dismiss warning signs and/or symptoms if they appear gradually and are unclear.
1	The first aider should not assume that the person exhibiting warning signs and/or symptoms is just going through a phase or misusing substances.
1	The first aider should not assume that the warning signs and/or symptoms of psychosis will go away on their own.
1	The first aider should be aware that the warning signs and/or symptoms of psychosis may vary from person to person and can change over time.
1	The first aider should exercise caution in how they react to or interpret potential warning signs as the person they are helping may not develop psychosis.
1	The first aider should take into consideration the spiritual and/or cultural context of the person's behaviours.
1	The first aider should attempt to determine whether the person's level of functioning has declined.
1	People developing a psychotic disorder will often not reach out for help. If the first aider is concerned about someone, they should approach the person in a caring and non-judgemental manner to discuss their concerns.
1	The first aider should try to find out what type of assistance the person believes will help them.
1	The first aider needs to convey that early intervention is important to prevent symptoms from escalating, without putting pressure on the person to seek help.
1	The first aider should reassure the person that it's okay to seek help and point out that seeking help is a sign of strength rather than a sign of weakness or failure.
1	The first aider should convey a message of hope to the person by assuring them that help is available and things can get better.
1	The first aider should know what services are available locally.
1	The first aider should find out as much as they can about psychosis, including recommended treatments and what services are available in the person's local area.
1	The first aider should encourage the person who is experiencing the symptoms of psychosis to be involved in the process of seeking information about psychosis as much as possible.
1	The first aider should try to determine whether the person has a supportive social network and if they do, the first aider should encourage them to utilise these supports.
1	The first aider should try to find some common ground for discussion, gradually building up towards more specific questions about the person's psychotic experiences.
1	Someone who is experiencing profound and frightening changes such as psychotic symptoms will often try to keep them a secret. The first aider should be aware that the person they are trying to help might not trust them or might be afraid of being perceived as "different", and therefore may not be open with them.
1	The first aider should state, in specific behavioural terms, why s/he is concerned about the person and should not speculate about their diagnosis.

1 The first aider should ask the person whether they have noticed changes in their behaviour. If they have noticed changes, the first aider should ask whether these changes are bothering them or whether they are distressed by their experiences.

1 The first aider should ask the person if they have felt this way before and if so, what they have done in the past that has been helpful.

1 The first aider should provide the person with information about local services.

1 If either the person experiencing psychosis or the first aider lacks confidence in the medical advice they have received, they should seek a second opinion from another medical or mental health professional.

1 If the person decides to seek professional help, the first aider should make sure that the person is supported both emotionally and practically in accessing services.

1 If the person decides to seek professional help, the first aider should encourage them to take a support person to their appointment.

1 As far as possible, the first aider should get the permission of the person before writing or speaking to professionals or others about them.

1 The first aider should always treat the person with respect.

1 The first aider should allow the person to talk about their experiences and beliefs if they want to.

1 As far as possible, the first aider should let the person set the pace and style of the interaction.

1 The first aider should understand the symptoms for what they are and should try not to take them personally.

1 The first aider should be honest when interacting with the person and should not make them any promises that cannot be kept.

1 The first aider should try to empathise with how the person feels about their beliefs and experiences, without stating any judgments about the content of those beliefs and experiences.

1 The first aider should avoid being intrusive as intense, interpersonal interactions can make psychotic symptoms worse.

1 The first aider should not use sarcasm when interacting with a person who may be experiencing psychosis.

1 The first aider should avoid using patronising statements when interacting with a person who may be experiencing psychosis.

1 The first aider should not touch the person without their permission.

1 The first aider should ask the person about what will help them to feel safe and in control.

1 Unless the person is a danger to themselves or others, the first aider should respect their privacy and right to confidentiality.

1 The first aider should allow the person to stay in control by offering choices of how s/he can help them where possible.

1 The first aider should encourage the person who is experiencing psychosis to talk openly about their experiences.

1 If the person is unwilling to talk with the first aider, the first aider should not try to force them to talk about their experiences.

1 If the person is unwilling to talk with the first aider, the first aider should let them know that s/he will be available if they would like to talk in the future.

1 The first aider should recognise that the person may be frightened by their thoughts and feelings.

1 The first aider should reassure the person that s/he is there to help the person and wants to keep them safe.

1 The first aider should understand that the person may be behaving and talking differently due to psychotic symptoms.

1 The first aider must recognise that the person who may be experiencing psychosis may find it difficult to tell what is real from what is not real.

1 The first aider should avoid confronting the person and should not criticise or blame them.

1 The first aider should make sure that s/he approaches the person privately about their experiences, in a place that is free of distractions.

1 The first aider should try to tailor their approach and interaction to the way the person is behaving (e.g. if the person is suspicious and is avoiding eye contact, the first aider should be sensitive to this and give the person the space they need).

1 The first aider should let the person know that s/he is there to support them.

1 The first aider should ask the person if they want to talk about how they are feeling.

1 The first aider should recognise that the delusions and/or hallucinations are very real to the person.

1 The first aider should not laugh at the person's symptoms of psychosis.

1 Until the first aider knows the content and context of the person's delusions, it is important to keep themselves safe from potentially aggressive reactions

1 The first aider should not dismiss, minimise or argue with the person about their delusions and/or hallucinations.

1 If the person exhibits paranoid behaviour, the first aider should not encourage or inflame the person's paranoia.

1 People experiencing symptoms of psychosis are often unable to think clearly. The first aider should respond to disorganized speech by communicating in an uncomplicated and succinct manner, and should repeat things if necessary.

1 After the first aider speaks, they should be patient and allow plenty of time for the person to digest the information and respond.

1 If the first aider doesn't understand something that they think is important to the conversation, they should ask the person to clarify it.

1 If the person is showing a limited range of feelings, the first aider should be aware that it does not mean that the person is not feeling anything.

1 The first aider should not assume the person cannot understand what they are saying, even if the person's response is limited.

1 The first aider should not act alarmed, horrified or embarrassed by the person's hallucinations or delusions.

1 In the event of a crisis, when the person experiencing psychosis has become acutely unwell...the first aider should try to remain as calm as possible

1 In the event of a crisis, when the person experiencing psychosis has become acutely unwell …the first aider should try to maintain safety and protect the person, themselves and others around them from harm.

1 In the event of a crisis, when the person experiencing psychosis has become acutely unwell …the first aider should allow as safe a physical distance between themselves and the person as practical to maintain interaction.

1 In the event of a crisis, when the person experiencing psychosis has become acutely unwell…the first aider should have access to an exit.

1 In the event of a crisis, when the person experiencing psychosis has become acutely unwell…the first aider should ask the person whether they would like her/him to decrease distractions (e.g. turn off TV, radio, dishwasher etc.) and lower stimulation (e.g. reduce room lights).

1 In the event of a crisis, when the person experiencing psychosis has become acutely unwell…the first aider should evaluate the situation by assessing the risks involved (e.g. whether there is any risk that the person will harm themselves or others).

1 In the event of a crisis, when the person experiencing psychosis has become acutely unwell…the first aider should use a moderate, non-threatening tone of voice.

1 In the event of a crisis, when the person experiencing psychosis has become acutely unwell…the first aider should speak quietly at a moderate pace and should answer all the person's questions calmly.

1 In the event of a crisis, when the person experiencing psychosis has become acutely unwell…the first aider should communicate in a clear and concise manner and use short, simple sentences.

1 In the event of a crisis, when the person experiencing psychosis has become acutely unwell …the first aider should remain aware that they may not be able to de-escalate the situation and if this is the case, they should be prepared to call for assistance.

1 In the event of a crisis, when the person experiencing psychosis has become acutely unwell…the first aider should assess whether the person is at risk of suicide.

1 In the event of a crisis, when the person experiencing psychosis has become acutely unwell…and the person is a danger to themselves or others, the first aider should make sure they are evaluated by a medical or mental health professional immediately.

1 In the event of a crisis, when the person experiencing psychosis has become acutely unwell…if other people arrive, the first aider should explain to the person experiencing psychosis who the people are, that they are there to help and how they are going to help.

1 In the event of a crisis, when the person experiencing psychosis has become acutely unwell …the first aider should try to limit access to objects that the person could use to harm themselves or others until crisis staff arrive.

1 In the event of a crisis, when the person experiencing psychosis has become acutely unwell …if crisis staff arrive, the first aider should convey specific, concise observations about the severity of the person's behaviour and symptoms to the crisis staff.

1 In the event of a crisis, when the person experiencing psychosis has become acutely unwell …if hospitalisation is required, the first aider should encourage the person to go voluntarily.

1 In the event of a crisis, when the person experiencing psychosis has become acutely unwell…if the person will not go to the hospital voluntarily, the first aider should be prepared to seek involuntary hospitalisation for the person only as a last resort.

1 In the event of a crisis, when the person experiencing psychosis has become acutely unwell …if the first aider's concerns about the person are dismissed by the services they contact, they should persevere in trying to seek support for the person.

1 In the event of a crisis, when the person experiencing psychosis has become acutely unwell…the first aider should assess whether it is safe for the person to be alone and if not, should ensure that someone stays with the person.

1 In the event of a crisis, when the person experiencing psychosis has become acutely unwell…the first aider should be aware that the person might act upon a hallucination or delusion.

1 In the event of a crisis, when the person experiencing psychosis has become acutely unwell …the first aider should remember that their primary task is to de-escalate the situation and therefore should not do anything to further agitate the person.

1 The first aider should be aware that people with psychosis are not usually aggressive and are at a much higher risk of harming themselves than others.

1 The first aider should not respond in a hostile, disciplinary or challenging manner to the person who is being aggressive.

1 The first aider should avoid asking too many questions as they can spark defensiveness and further anger.

1 The first aider should not threaten the person as this may increase fear or prompt aggressive behaviour.

1	The first aider should remain aware that the person’s symptoms or fear causing the aggression may be exacerbated by the first aider taking certain steps (e.g. involving the police).
1	The first aider should take any threats or warnings seriously, particularly if the person believes they are being persecuted.
1	The first aider should not try to restrict the person’s movement (e.g. if the person wants to pace up and down the room).
1	The first aider needs to recognise that certain symptoms of psychosis (e.g. visual or auditory hallucinations) can cause people to become aggressive.
1	If the first aider is frightened, they should seek outside help immediately as they should never put themselves at risk
1	When contacting the appropriate service, the first aider should not assume the person is experiencing a psychotic episode but should outline any symptoms and immediate concerns.
1	If the person’s aggression escalates out of control at any time, the first aider should remove themselves from the situation and call the police.
1	If the person’s aggression escalates out of control at any time, the first aider should remove themselves from the situation and call the crisis team.
1	If the police are called, the first aider should tell them that the person is experiencing a psychotic episode and that the first aider needs the help of the police to obtain medical treatment and to control the person’s aggressive behaviour.
1	The first aider should let the police know whether or not the person is armed.
1	The first aider should know how to de-escalate the situation if the person they are trying to help becomes aggressive.
1	If necessary, the first aider should remove any weapons or objects that could be used as weapons from the person’s immediate environment.
1	If the person is showing aggression, the first aider should avoid raising their voice and should not talk too fast.
1	If the person is showing aggression, the first aider should stay calm and avoid nervous behaviour (e.g. shuffling their feet, fidgeting, making abrupt movements).
1	The first aider should be aware that the person who is experiencing psychotic symptoms may lack insight that they are unwell.
1	If the person is unwilling to see a doctor or mental health professional, the first aider should explore the reasons why the person is unwilling to seek help.
1	A lot of people are hesitant to seek treatment for psychosis because they are afraid of being hospitalised. If possible, the first aider should reassure the person that if treatment is started early enough, hospitalisation might not be necessary.
1	The first aider should emphasise to the person that medication could make a huge difference in the way they are feeling.
1	The first aider should stress the potential benefits of getting help.
1	The first aider should assist by giving the person resources (e.g. pamphlets, telephone numbers for services).
1	If the person does lack insight, the first aider should be aware that they might actively resist the first aider’s attempts to encourage them to seek help.
1	The first aider should provide a consistent message encouraging the person to seek help.
1	Because symptoms of mental illness may stem from other physical illnesses, the first aider should initially encourage the person to see their doctor for a check-up, rather than suggesting from the start that it may be psychosis.
1	The first aider should not use deception to get the person into a health professional’s office (e.g. that the person should get their backache checked out).
1	If the person refuses to get help, the first aider should remain friendly and open to the possibility that they may want the first aider’s help in the future.

1	The first aider should remain patient, as people experiencing psychosis often need time to develop insight regarding their illness.
1	When someone who is experiencing symptoms of psychosis denies that they are unwell, the first aider's course of action should depend on the type and severity of the person's symptoms.
1	The first aider should consult a mental health professional for advice on how to help the person.
1	If the person is taken to hospital, the first aider should ask to speak with the medical professional conducting the person's assessment. The first aider will then be able to explain the symptoms that have been occurring as people experiencing psychosis who do not want treatment may hide their behaviour or ideas from a professional.
1	The first aider needs to recognise that unless a person with psychosis meets the criteria for involuntary committal procedures, they cannot be forced into treatment.
1	The first aider should never threaten the person with the mental health act or hospitalisation.
1	The first aider needs to be aware that the person may be experiencing transient symptoms that will resolve with time or with a reduction in current stressors.
1	Unless the person is a danger to themselves or others, the first aider should respect the person's right not to seek help.
1	The first aider should not try to convince the person that they are psychotic.
1	If the person refuses to seek help, the first aider should encourage them to talk to someone they trust.
1	The first aider should recognise that even if the person does realise that they are unwell, their confusion and fear about what is happening to them may lead them to deny that there is anything wrong.
2	The first aider should be aware of what constitutes culturally appropriate behaviours so that they don't misinterpret such behaviours as symptoms of psychosis (e.g. in some communities, limited eye contact is expected behaviour).
2	If the first aider gives the person information about psychosis, they should remain aware that some Aboriginal People cannot read well and may need assistance with pamphlets and books.
2	The first aider should be aware of Aboriginal Health Services and the roles of elders and/or healers in the community.
2	If possible, the first aider should prepare the person for what they might expect when they go to the doctor or mental health professional.
2	If the first aider is not a family member, they should ask the person whether it's okay for them to talk to the person's family.
2	The first aider should learn what the best practice is for psychosis and be prepared to be assertive and persistent in seeking appropriate care for someone who may have psychosis.
2	If the person has sought professional help, the first aider should ask them if they understood what was said to them in order to clarify any medical jargon used.
2	The first aider should be mindful not to use jargon and should keep their language simple and applicable to the local community.
2	If the person is very fearful, the first aider should keep them company to reassure them that they are not alone.
2	The first aider should acknowledge that although s/he realises that the hallucinations and/or delusions are very real to the person, s/he does not hear, see, smell, or feel what the person is experiencing.
2	The first aider should not enter into lengthy discussions with the person about the content of their delusions or hallucinations.

2	The first aider should ask questions about the content of the person's delusions, particularly any elements that indicate the potential for harming themselves or others.
2	
2	In the event of a crisis, when the person experiencing psychosis has become acutely unwell…if the person has an advance directive/relapse prevention plan, the first aider should follow the guidelines set out in the plan.
2	In the event of a crisis, when the person experiencing psychosis has become acutely unwell…the first aider should move to quieter surroundings with the person if practical.
2	In the event of a crisis, when the person experiencing psychosis has become acutely unwell…the first aider should not block the person's exit.
2	In the event of a crisis, when the person experiencing psychosis has become acutely unwell …the first aider should decrease distractions (e.g. turn off TV, radio, dishwasher etc.) and lower stimulation (e.g. reduce room lights) if possible.
2	In the event of a crisis, when the person experiencing psychosis has become acutely unwell…the first aider should try to find out if the person has anyone s/he still trusts (e.g. close friends, family) and should try to enlist their help.
2	In the event of a crisis, when the person experiencing psychosis has become acutely unwell…the first aider should explain to the person why they believe that a professional assessment is necessary.
2	In the event of a crisis, when the person experiencing psychosis has become acutely unwell…the first aider should get medical help for the person as quickly as possible and explain to the person why they believe this is necessary.
2	In the event of a crisis, when the person experiencing psychosis has become acutely unwell…if hospitalisation is required and the person will not go voluntarily, the first aider should see if one of the person's relatives or friends can persuade them to go to hospital.
2	In the event of a crisis, when the person experiencing psychosis has become acutely unwell…if the person needs to be hospitalised, the first aider should support them by focusing conversation on how a hospital stay will bring relief through reducing psychotic symptoms.
2	In the event of a crisis, when the person experiencing psychosis has become acutely unwell…the first aider needs to be aware of the cultural consequences that hospitalisation may have for the person and their community.
2	In the event of a crisis, when the person experiencing psychosis has become acutely unwellThe first aider should never try to physically restrain someone who is experiencing psychosis.
2	If the first aider is alone with the person experiencing psychosis, they should contact someone to come and stay with them until professional help arrives.
3	The first aider should be aware of the different conceptualisations of psychosis and the terminology used to describe these concepts in Aboriginal communities.
3	The first aider should let the person vent their frustration in a way that does not threaten others.

Table 3 - Endorsed statements from the Delphi study "First Aid for Suicidal thoughts and behaviours"

Round endorsed	Endorsed Statements
1	The first aider should learn about the behaviours that are considered warning signs for suicide in the person's community.
1	If the first aider thinks someone might be having suicidal thoughts, they should ask that person directly.

1 The first aider should find out if the suicidal person has already taken steps to secure the means to end their life.

1 The first aider should ask the suicidal person if they have been using drugs or alcohol.

1 The first aider should ask the suicidal person if they have ever made a suicide attempt in the past.

1 It is important for the first aider to discuss the issue of suicide directly and not avoid use of the word 'suicide'.

1 The first aider should allow the suicidal person to discuss their feelings.

1 The first aider should re-assure the person that they may feel better once they have spoken about their problems.

1 The first aider needs to allow the suicidal person to talk about their reasons for wanting to die.

1 The first aider should understand that the threat of suicide may indicate that a person is trying to communicate to the first aider how badly he or she feels. The first aider should not express negative judgement about the person, their thoughts or intentions.

1 The first aider should never dare the person to take their own life.

1 The first aider should acknowledge the person's courage in talking about their suicidal feelings.

1 The first aider should never ignore the person's thoughts or intentions of suicide.

1 If the first aider thinks that the person is thinking about suicide they should act immediately.

1 The first aider should be aware of the range of people who are available to help the person.

1 The first aider should allow the person to suggest someone they would like and would trust to help support them while they get better.

1 The first aider should help the person find support from someone in their community. This may include: family or friends, a community Elder, Aboriginal or mainstream health worker, Aboriginal or mainstream mental health worker, community health care centre, support groups, religious minister, telephone counselling service, school counsellor, youth group leader, or sporting coach.

1 The first aider should ensure that the person gets help from an appropriate professional or someone in the community who can support them until the crisis resolves.

1 The first aider must not leave someone who is feeling suicidal on their own.

1 The first aider should do something to help comfort the person such as sitting with them, making them a cup of tea/coffee, offering them time, friendship and encouragement.

1 The first aider should be aware of the range of intervention/management options available to the person. These may include: counselling or clinical treatment, increasing family, community or professional support.

1 The first aider should discuss with the person the range of intervention/management options available.

1 The first aider should get the person to think about ways they have coped in the past.

1 The first aider should focus on the person's strengths.

1 The first aider should encourage the suicidal person to avoid excessive use of drugs and/or alcohol.

1 A first aider must never agree to keep the risk of suicide a secret.

1 If the first aider can't get the suicidal person to agree to hand over the means of suicide (for example, pills, gun, razors), emergency services must be contacted immediately.

1 The first aider must take steps to ensure the suicidal person will receive medical and/or psychological help once the immediate crisis has passed.

1 The first aider should not make promises that they cannot keep.

1	The first aider needs to find out what has supported the suicidal person in the past, and whether these supports are still available.
2	The first aider should be aware that sometimes suicide is well-planned and sometimes it is impulsive.
2	The first aider should be aware that if the person is intoxicated with drugs or alcohol, it may increase their risk of suicide.
2	The first aider should be aware that personal relationship breakdown can lead to an increased risk of suicide.
2	The first aider should be aware that people who are thinking about suicide may stop talking to their family and friends.
2	The first aider should encourage the person to talk about their feelings with someone they trust such as a friend or family member, Aboriginal worker, an Aboriginal mental health worker, an Elder, or a community liaison officer.
2	If the person says that they are suicidal, the first aider must take them seriously.
2	Whether the person is drunk or sober, if the person says that they are suicidal, the first aider must take them seriously.
2	The first aider should be aware that the person may not admit to suicidal feelings because they feel shame.
2	The first aider should establish whether the person has definite plans and intentions to take their life as opposed to vague suicidal notions such as "what's the point," or "I can't be bothered going on".
2	The first aider should ask the suicidal person if they have a plan for suicide.
2	The first aider should ask the suicidal person how they intend to suicide.
2	The first aider should ask the suicidal person if they have ever known anyone who has died by suicide.
2	The first aider should not argue with the person about their thoughts of suicide.
2	If the suicidal person refuses to involve someone else, the first aider must contact a professional.
2	In order to reduce the risk of the person taking their life, the first aider should discuss options that may assist the person to cope, without making them feel judged.
2	The first aider can help work out ways of dealing with difficulties that seem impossible to overcome by discussing the suicidal person's specific problems.
2	If the person is suicidal and the first aider cannot secure their means to suicide, then emergency help must be sought immediately.
2	The first aider should plan with the person the actions that they can take together to keep the person calm and safe until professional help arrives.
2	The first aider should engage the help of a mental health professional, friends, family, Indigenous health workers or Elders to guide the person through the crisis.
2	The first aider must uphold the person's right to confidentiality, unless the first aider is worried about the person's risk of harm to self or others.
2	The first aider should offer the person 24-hour safety contacts in case the person feels unable to continue (such as a suicide helpline, professional helper or family member).
3	The first aider must have the person's permission before they seek help from other members of the community.
3	The first aider should be aware that some communities do not find the term 'suicide' acceptable. If this is the case, the first aider should try to use alternative words.

Table 4 - Endorsed statements from the Delphi study "First Aid for Deliberate self-injury"

Round endorsed	Endorsed Statements
1	The first aider should learn about the behaviours that are considered warning signs for self-injury in the person’s community.
1	The first aider should express their concern.
1	The first aider should remain calm, and avoid expressions of shock or anger.
1	The first aider should intervene in a supportive and non-judgemental way.
1	The first aider should ask the person if they are suicidal.
1	If the person has injured themselves by taking an overdose of medication or consuming poison, the first aider should call an ambulance as the risk of permanent harm or death is high.
1	The first aider should try to avoid a strong negative reaction to the self-injury and discuss it calmly with the person.
1	The first aider should understand that self-injury is a coping mechanism.
1	The first aider should encourage the person to talk about the feelings motivating the self-injury.
1	The first aider should encourage the person to speak to someone they trust next time they feel the urge to self-injure.
1	The first aider should encourage the person to come and have a yarn about their feelings instead of self-injuring.
1	The first aider should encourage the person to draw or write about their distress instead of engaging in self-injury.
1	The first aider should seek professional mental health care for the person they are helping if they have expressed a desire to die, or doesn’t care if they live or die.
1	The first aider should call an ambulance regardless of the person’s wishes if the injury is life-threatening, such as arterial bleeding.
1	The first aider should encourage the person to seek professional help.
1	The first aider should help the person access appropriate help from a social and emotional wellbeing counsellor or worker.
2	If the person is receiving professional mental health care, or has been in contact with a social and emotional wellbeing counsellor/worker, the first aider should ask if the professional knows about the injuries.
2	The first aider should be aware that deliberate self-injury, such as cutting and burning, is fundamentally different to culturally accepted Aboriginal ceremonial or grieving practice.
2	n order to distinguish between cultural practice and deliberate self-injury, the first aider needs to be aware of the local cultural practices.
2	In order to distinguish between cultural practice and deliberate self-injury, the first aider should engage the help of local Aboriginal health workers.
2	The first aider should intervene to try and stop the person from deliberately injuring themselves, but only if it does not put the first aider or others at risk of harm.
2	The first aider should ask whether they can do anything to alleviate the distress.
2	The first aider should keep in mind that ‘stopping self-injury’ should not be the focus; instead the first aider should look at ways to relieve the person's distress.
3	The first aider should be aware that deliberate self-injury is a risk factor for suicide in the future.
3	The first aider should call an ambulance regardless of the person’s wishes if the person has injured their eyes.

Table 5 - Endorsed statements from the Delphi study “Cultural considerations and communication techniques”

Round endorsed	Endorsed Statements
1	The first aider should stay with the person until professional help arrives.
1	The first aider should not ignore any injuries, but instead acknowledge to the person that they have noticed them.
1	The first aider should encourage the person to seek professional help.
1	The first aider should ensure the person knows about and has access to some form of professional care that is right for them, in case they feel the urge to self-injure again in the future.
1	In order to distinguish between cultural practice and deliberate self-injury, the first aider should ask the person why they are injuring themselves.
1	The first aider should seek professional mental health care for the person, even if their injuries do not require medical treatment.
1	The first aider should learn about the specific cultural beliefs that surround mental illness in the person's community
1	The first aider should consider that Aboriginal people understand mental health within a wider context of health and wellbeing which includes concepts of social and emotional functioning.
1	The first aider should be aware that Aboriginal people may see behaviour signs of mental illness as part of a person's spirit or personality and may not conceptualise them as a form of treatable mental illness.
1	The first aider should be aware of the concept of mental illness, (the symptoms, behaviour and terminology) that is used by the person's community.
1	The first aider should take into consideration the spiritual and/or cultural context of the person's behaviours.
1	The first aider should be aware of the cultural concept of shame within the person's community.
1	The first aider should have a yarn with the person, spend time with them and let them know that they are worried about them.
1	The first aider should ask the person where they would be most comfortable to have a yarn; confined places may cause the person anxiety but outdoors on verandas or near trees might be more relaxing.
1	The first aider should be sensitive to the cultural norms of the person's community, such as eye contact and seating position.
1	The first aider should avoid too much eye contact as it can make the person feel as though she/he is being stared at or judged, especially if discussing topics that may cause the person to feel shame.
1	The first aider should use simple and clear language.
1	The first aider should re-assure the person that they may feel better once they have spoken about their problems.
1	The first aider should ask the person's permission before asking questions about sensitive topics.
1	The first aider should allow periods of silence while the person considers their response to a question.
1	The first aider should give the person time to tell their story.
1	The first aider should never make the person feel shame.
1	The first aider should ask the person's permission before speaking to family members.
1	The first aider should avoid asking questions that might embarrass the person in front of their family or friends.
2	The first aider should be aware that it is common for the experiences of Aboriginal people (such as seeing spirits or hearing voices of recently deceased loved ones) to be misdiagnosed or mislabelled as mental illness, when they are not in fact ill.

2 The first aider should be aware that the misdiagnosis of Aboriginal people can be a barrier to help-seeking.

2 The first aider should understand how an individual might feel shame; that is by discussing topics or performing behaviours that may be considered mad, abnormal, unusual or embarrassing.

2 The first aider should understand how a community might feel shame; for example being asked or forced to do things not in the normal community way; or by keeping contact with someone who is acting unusual because of mental illness.

2 The first aider should understand how Aboriginal people might feel societal shame; for example as a result of historical factors such as dispossession of Aboriginal land and domination of culture.

2 The first aider should be aware of the way shame affects Aboriginal behaviour; for instance, some Aboriginal people may be afraid of attending a mainstream hospital because, historically, being admitted to a hospital with a mental illness caused shame on family and community.

2 The first aider should be aware that simply reading these guidelines will not equip them to be competent in providing assistance to Aboriginal people.

2 The first aider should be aware that these cultural consideration guidelines are not exhaustive.

2 The first aider should be aware that what is a respectful way to communicate with an Aboriginal person, including body language, seating position and use of certain words, may differ from community to community, and region to region (especially between rural and remote areas).

2 The first aider should try to sit facing the same way as the person, looking out rather than directly facing them.

2 If the person is suspicious or expresses resentment towards the first aider because they are from a different culture, the first aider should empathise with the person and reassure them that they are there to help.

2 The first aider should limit closed questions which lead to single word (eg. yes/no) answers. For example: Do you feel unwell?

2 If the first aider is worried about the person's safety, or the person is experiencing a crisis, then the first aider should ask the person’s family to get involved in supporting them while they get better.

2 The first aider must uphold the person's right to confidentiality.

2 The first aider should have a yarn with the person about how they would like to be helped.

2 If the person finds it too hard to talk about their problems, the first aider should respect that.

2 The first aider should be aware that establishing a network of support for an Aboriginal person is a very important step in helping them resolve their mental health crisis, especially if access to professional support, or mental health services, is limited.

2 The first aider should encourage the person to build personal relationships with people who they can trust, respect as a mentor, and ask for care and assistance when feeling unwell.

2 If the first aider is providing mental health first aid outside their own community then they should be culturally competent and practice cultural safety.

2 The first aider should encourage the person to build personal relationships with a police officer or youth worker at a community centre, an Aboriginal mental health worker, an Elder, or community liaison officer.

2 The first aider should encourage the person to participate in activities that will help them to develop feelings of purpose, belonging and achievement.

2 The first aider should be aware that it is more important to make the person feel comfortable, respected and cared for, than to do all the 'right things' and follow all the 'rules' when communicating with an Aboriginal person.

2 The first aider should not speak to the person in a patronising manner.

2	The first aider should tell the person that they care and want to help.
2	If the family of the person are present, the first aider should expect that they might answer some questions on behalf of the person.
2	The first aider should avoid asking lots of questions.
2	The first aider should not criticise members of the extended family.
2	The first aider should not falsely imply that by talking about mental illness or mental health problems, the person’s problems will go away.
2	In order to encourage the person to participate in positive activities, the first aider should discuss with the person what the person's interests and activities are.
2	If the first aider is worried about the person's safety, or the person is experiencing a crisis, then the first aider should be persistent in trying to get the person help and support from others.
2	The first aider must have the person's permission before they seek help from other members of the community, unless they are worried about the person's risk of harm to self or others.
3	Because family and friends are a very big part of Aboriginal culture, the first aider should expect involvement by family/friends in caring for the person.
3	The first aider should ask if the person would feel more comfortable talking away from family or friends.

Table 6 - Endorsed statements from the Delphi study “First Aid for Trauma and loss”

Round endorsed	Endorsed Statements
1	The first aider should be aware that all recognition and treatment of mental distress within Aboriginal communities should involve an understanding of trauma, grief and loss.
1	The first aider should recognise that trauma, grief and loss are seen as major contributing factors to mental distress within Aboriginal communities.
1	The first aider should understand that the impact of trauma and grief on Aboriginal peoples’ mental health is related to the history of invasion, the ongoing impact of colonisation, loss of land and culture, racism within the wider Australian community, high rates of premature mortality, family separations, incarceration and deaths in custody.
1	The first aider should understand that when trauma is ignored and there is no support for dealing with its effects, trauma can be passed from one generation to the next. This process is often called intergenerational trauma.
1	The first aider should understand what intergenerational trauma is.
1	The first aider should be aware that because Aboriginal communities are highly integrated, bereavement and traumatic events often have a widespread and devastating impact on health and community stability, even when only a few people are the primary victims (e.g. in a car accident).
1	The first aider should be aware that the high rates of early mortality in Aboriginal communities means that Aboriginal people are likely to experience the deaths of loved ones at an early age, and frequently throughout their lifetime.
1	The first aider should be aware that where death, separation and loss are more frequent, a person may find it increasingly difficult to deal with each individual loss.
1	The first aider should be aware of the effects that past trauma can have on current trauma for the person.
1	The first aider should be aware that if a person has experienced past trauma, even if it was a very long time ago, the event can still affect their lives.
1	The first aider should be aware that for many Aboriginal people, participating in traditional ceremonies, such as ‘sorry business’, is very important in the

resolution of grief or trauma.

1 The first aider should be aware that a traumatic experience may cause the person to become suicidal.

1 The first aider should determine whether or not it is safe to approach the person before taking any action (for example, danger from fire, weapons or debris).

1 The first aider should explain to the person what their role is and why they are present.

1 The first aider should try to create a safe environment.

1 If the person is badly injured, the first aider should encourage the person to go to a hospital.

1 The first aider should try to remain calm.

1 The first aider should stay with the person.

1 The first aider should speak clearly and avoid clinical and technical language.

1 The first aider should communicate with the person as an equal, rather than as a superior or expert.

1 The first aider should try to show that they understand and care.

1 The first aider should remember that behaviour such as withdrawal, irritability and bad temper may be a response to the trauma, and they should avoid taking such behaviour personally.

1 The first aider should reassure the person that their reactions are normal responses to abnormal circumstances, by saying something like “It’s okay to be feeling the way you are feeling”.

1 The first aider should encourage the person to talk about their feelings when the person feels ready to do so.

1 If the person does not wish to talk to the first aider, they should encourage the person to consider calling a crisis line, going to a crisis centre, or using other community resources.

1 The first aider should remember that they are not the person's counsellor.

1 The first aider should avoid using clichés like "you must be strong" and "life goes on".

1 The first aider should be aware that experiences of trauma, grief and loss affect the whole person (mind, spirit, body) and their relationships with others.

1 The first aider should be aware that trauma, grief and loss become a problem when the person is overwhelmed by their feelings and is unable to move on with their life.

1 The first aider should be aware that if trauma, grief and loss become a problem, the person should be encouraged to seek professional help.

1 The first aider should be aware that because Aboriginal people are likely to experience loss in the context of traumatic or untimely circumstances, they are also likely to experience overwhelming reactions to feelings of trauma, grief or loss.

1 The first aider should be aware of what cultural practices are used for trauma, grief or loss within the person’s community.

1 The first aider should be aware that it is not unusual for Aboriginal people to see, hear or talk to the spirits of their deceased loved ones.

1 The first aider should be sensitive to the cultural practices of the person’s community so they do not cause the person embarrassment or shame. For instance, some communities avoid referring to a deceased loved one by name.

1 The first aider should be aware that, if the trauma has involved the death of a loved one, the person may find anniversaries, celebrations (such as Christmas or birthdays) or hearing about situations that remind them of the loss, stressful.

1 The first aider should allow for moments of silence and reflection.

1 The first aider should not interrupt the person.

1 The first aider should give the person lots of time to tell their story.

1 The first aider should listen to the person in a non-judgemental and accepting way.

1 The first aider should not force the person to tell their story or probe for more details.

1 If at any time the first aider feels they cannot listen to the details of the trauma, the first aider needs to let the person know, while offering support and understanding.

1 The first aider should not be afraid to admit they 3 what to say if this is the case.

1 The first aider should avoid saying things that minimise the person's experience, such as "you should just be glad you're alive."

1 The first aider should not tell the person how they "should" be feeling.

1 The first aider should not make promises they can't keep.

1 The first aider should not say to the person 'I know how you feel'.

1 The first aider should let the person know that it is ok for the person to express their grief, even though it may be hard for the first aider to see them so upset.

1 If the person believes they have been visited by the spirit of their loved one, the first aider should reassure them that this is a common experience for many Aboriginal people, and encourage them not to be frightened or feel shame.

1 The first aider should help the person by simply being there for them.

1 The first aider should be available and attentive to the person.

1 The first aider should let the person know that they care.

1 The first aider should allow the person to make their own decisions.

1 The first aider should avoid judging, blaming or nagging the person.

1 The first aider should avoid repeatedly asking for details of the trauma.

1 The first aider should not expect that the person will be 'over it' within a few weeks.

1 The first aider should be patient with the person.

1 If the person is experiencing changes in their mood, feelings of grief, loss or energy, the first aider should reassure the person that it is common to have good and bad days.

1 If the person feels guilty or worried about being happy, the first aider should reassure them that it is ok for them to enjoy themselves and the company of others.

1 The first aider should encourage the person to identify sources of support such as loved ones and friends.

1 The first aider should respect the person's need to be alone at times.

1 The first aider should encourage the person to fulfil their particular cultural practices for dealing with trauma, grief or loss. For example, going home to country and participating in sorry business.

1 The first aider should provide the person with information and resources about dealing with trauma, grief or loss.

1 The first aider should encourage the person to allow themselves to feel sadness and grief over what has happened.

1 The first aider should encourage the person to express their feelings when they need to.

1 The first aider should not discourage the person from expressing their feelings of trauma, grief or loss.

1 The first aider should encourage the person to express their trauma, grief or loss in ways that do not cause harm to self or others.

1 The first aider should encourage the person to express their trauma, grief or loss in ways that are meaningful to them. Some examples could include physical activity, music, writing or journaling, art, praying or meditating, story telling, cultural activities or ceremonies.

1 The first aider should respect the person's right to confidentiality by not telling others what the person has told them without the person's permission.

1 The first aider should help the person develop a list of people, services, or places to contact when the going gets tough.

1 The first aider should encourage the person to learn effective coping strategies.

1 The first aider should encourage the person to think about and use coping strategies that have helped in the past.

1 The first aider should encourage the person to take care of themselves.

1 The first aider should encourage the person to find ways to relax.

1 The first aider should encourage the person to practice slow deep breathing.

1 The first aider should encourage the person to either take time out from normal activities, or maintain their routine, depending on what feels best to the person.

1 The first aider should encourage the person to eat well and regularly.

1 The first aider should encourage the person to get regular exercise.

1 The first aider should encourage the person to do things that feel good to them (eg. take a walk, go fishing, watch television).

1 The first aider should encourage the person to get plenty of rest when they are tired.

1 The first aider should encourage the person to spend time somewhere they feel safe and comfortable.

1 The first aider should discourage the person from using negative coping strategies such as working too hard, using alcohol and other drugs, or engaging in self-destructive behaviour.

1 The first aider should encourage the person to be patient with themselves.

1 If at any time the person becomes suicidal, the first aider should encourage them to seek professional help.

1 The first aider should encourage the person to seek professional help if the person’s post-trauma symptoms are interfering with their usual activities for four weeks or more

1 The first aider should encourage the person to seek professional help if the person feels very upset or fearful for four weeks or more

1 The first aider should encourage the person to seek professional help if the person is overwhelmed by intense or distressing feelings at any time.

1 The first aider should encourage the person to seek professional help if the person’s important relationships are suffering as a result of the trauma at any time

1 The first aider should encourage the person to seek professional help if the person abuses alcohol or other drugs to deal with the trauma at any time

1 The first aider should encourage the person to seek professional help if the person feels jumpy or has nightmares relating to the trauma at any time

1 The first aider should encourage the person to seek professional help if the person can't stop thinking about the trauma for four weeks or more

1 The first aider should encourage the person to seek professional help if the person feels like no-one understands them at any time

1 The first aider should encourage the person to seek professional help if the person starts picking arguments with friends or getting into fights at any time

1 If the person is afraid that a deceased loved one's spirit is visiting them, the first aider should encourage the person to seek appropriate help (eg. Aboriginal health worker, Nungkari/Healer, Elder or family member).

1 If the person is afraid that they will suffer from payback for their loved one's death the first aider should encourage the person to seek appropriate help (eg. Aboriginal health worker, Nungkari/Healer, Elder, family member, or someone strong within their community that can help them with the issue).

1 The first aider should be aware that each person's needs will be different. Some may want one-on-one contact with a professional, while others may prefer participating in a group.

1 The first aider should know that for some people more traditional methods of healing, such as that provided by a Ngungkari/Healer will be important, while for others conventional grief counselling will be more helpful.

1 The first aider should be aware of the professionals that may be of help to the person (eg. psychologists, Bringing Them Home counsellors who specialise in stolen generations issues, healing circles or cultural healing groups, bereavement support groups, doctors, or religious leaders).

1 The first aider should be aware of the range of specialist services that provide help and assistance for different types of trauma. These include Centres Against Sexual Assault (CASA), counselling for victims of crime, Link-Up, Bringing Them Home counsellors etc.

1 The first aider should have a yarn with the person about their options for traditional, counselling and/or medical treatment.

1 The first aider should allow the person to make decisions about what sort of help will be best for them.

1 The first aider should assist the person to find professional help that the person can trust and feel comfortable talking to.

1 The first aider should encourage the person to find a professional who will encourage the person to tell their story.

1 The first aider should assist the person in finding out what help is available for trauma, grief or loss.

1 The first aider should be aware that trauma counselling suitable for Aboriginal people, may be uite difficult to find or to gain access to, as there is a shortage of appropriately trained Aboriginal psychologists and counsellors.

1 If the person has experienced, or is experiencing ongoing trauma (eg. past child abuse or family violence) the first aider should reassure the person that it is never too late to benefit from professional help.

1 The first aider should tell the person that reaching out for help is not a sign of weakness, but a normal need for people who are dealing with trauma, grief or loss.

1 The first aider should reassure the person that there is no need to feel shame about having a yarn with a doctor or health worker to help them through their healing.

1 The first aider should reassure the person that they may benefit from professional help.

1 If the person is feeling isolated, the first aider should reassure them this is a normal response after to the suicide of a loved one.

1 The first aider should encourage the person not to lay blame for the suicide.

1 The first aider should avoid placing blame or trying to explain the suicide.

1 The first aider should reassure the person that it is OK to talk about violence or abuse.

1 If the person discloses information about violence or abuse, the first aider should believe what the person tells them has happened.

1 The first aider should be aware that family violence is not part of Aboriginal culture.

1 The first aider should be aware that family violence is illegal and damaging for those affected.

2 The first aider should be aware that family violence involves a number of different forms of abuse: physical, verbal, emotional, sexual, or cultural.

2 The first aider should be aware that family violence affects all members of a family, including babies and children.

2 If any child discloses abuse, the first aider should contact the appropriate authorities.

2 The first aider should ask the person if they would like the first aider to remain with them.

2 The first aider should tell the person that everyone has their own pace for dealing with trauma.

2 The first aider should remember that providing support doesn't have to be complicated, and can involve small things like spending time together, having a cup of tea or coffee, chatting about day-to-day life or giving them a hug.

2 The first aider should avoid saying things that minimise the person’s feelings, such as “don’t cry” , “calm down” or “get over it”.

2 The first aider should ask the person how they would like the first aider to help them.

2 The first aider should encourage the person to identify sources of support, such as community members, support groups, men’s groups or women’s groups.

2 The first aider should be aware that the person may feel shame as a result of the suicide of a loved one.

2 The first aider should demonstrate cultural respect when assisting someone who has experienced trauma, grief or loss.

2 The first aider should be tolerant of any strong emotion expressed by the person, except where the person is threatening, abusive or violent.

2 The first aider should tell the person that they can talk to them another time if they don't want to do it now.

2 The first aider should encourage the person to tell others when they need or want something, rather than assume others will know what they want.

2 The first aider should encourage the person to seek professional help if the person is unable to enjoy life at all as a result of the trauma at any time.

2 The first aider should encourage the person to find a counselling service specialised in assisting with the person’s experience of trauma (eg. Stolen generations or bereavement counselling).

2 If the person has been a victim of crime, the first aider should consider the possibility that forensic evidence may need to be collected (for example, swabs from body, evidence on clothing or skin).

2 If the person has been a victim of crime, the first aider should be aware that the person may be very fearful of contacting police because of mistrust of police, possible family feuding or 'payback'.

2 The first aider should encourage the person not to let small day-to-day hassles build up and add to their stress.

2 The first aider should encourage the person to see a professional who is trained and has experience in treating Aboriginal people and their experiences of trauma, grief and loss.

2 The first aider should reassure the person that a lot of people need professional help while they are grieving or after experiencing trauma.

2 The first aider should be aware that the person might not be as distressed about the trauma as could be expected.

2 The first aider should acknowledge the person’s loss or trauma and what it means to them.

2 The first aider should be aware that it is not unusual for men to not want to express their feelings verbally, or to avoid having to ‘talk it out’.

2 If the trauma involved the death of a loved one, and the person thinks that they might have seen their loved one, or talks about them as if they are still alive, the first aider should reassure the person that this is normal.

2 The first aider should offer to assist the person to access professional help.

2 If the person has been a victim of crime, the first aider should suggest to the person that even if the person does not want to consider the possibility of pressing charges now, collecting evidence may be a good idea.

3 The first aider should be aware that the person might need some time to prepare (hours, days or weeks) before they feel ready to report any violence or abuse to the police.

3 The first aider should be aware that where there is ongoing trauma of any kind, the sooner professional help is sought the better.

3 The first aider must respect the person's right to refuse counselling, medical treatment and police intervention.

3 The first aider should tell the person that excessive intake of alcohol or other drugs is not likely to help the person.

3	If the person is reluctant to contact police, the first aider should help the person try to overcome their fears about police intervention. For instance, the first aider could offer to find out who the local Aboriginal liaison officer is, offer to stay with the person while they talk with police, or talk with the person about victims of crime compensation.
3	If the person is reluctant to contact police, the first aider should help the person try to overcome their fears about police intervention. For instance, the first aider could offer to find out who the local Aboriginal liaison officer is or offer to stay with the person while they talk with police.

Table 7 - Strongly rejected items that were rated as either Unimportant or Should not be included by 50% or more of the panel

Statement	Un-important	Should not be included	U + S
Depression Round 1			
If the first aider feels frustrated, s/he should let the depressed person know s/he is frustrated with the person's illness, not with them.	10	50	**60.0**
The first aider should complain a little about their life to the depressed person to help the person feel less alone.	5	80	**85.0**
The first aider should acknowledge how the person's depression affects her/him by sharing her/his feelings and concerns with the person.	10	40	**50.0**
If the depressed person refuses to seek or accept professional help, the first aider should use deception, coercion, threats, or whatever is necessary to ensure professional help is received.	0	90	**90.0**
The first aider should recommend that the person's depression is labelled as a "family problem" and that the whole family attends therapy. This will take the focus off the depressed person.	0	63.2	**63.2**
The first aider should ask the suicidal person if they are really serious or just looking for attention.	20.8	50	**70.8**
Suicide Round 1			
In order to reduce suicide risk, it is important for the first aider to try to solve the suicidal person's problems.	8.3	45.8	**54.1**
If the suicidal person has a weapon, the first aider should try to take it away from them.	8.3	45.8	**54.1**
If the suicidal person is consuming drugs or alcohol, the first aider should try to take it away from them.	4.2	45.8	**50.0**
If the first aider can't get the person to agree to hand over the means of suicide (for example, pills, gun, razors) they should try to take these things secretly.	4.2	83.3	**87.5**
The first aider shouldn't remove the means of suicide available to the suicidal person.	0	87.5	**87.5**
To help the suicidal person change their mind, the first aider should tell them they will ruin the lives of others if they die by suicide.	0	70.8	**70.8**
To help the suicidal person change their mind, the first aider should tell them they will go to hell if they die by suicide.	0	100	**100.0**

The first aider should tell the suicidal person that the feelings they are experiencing are caused by a mental illness.	0	91.7	**91.7**
The first aider should help the suicidal person to put their problems into perspective by reminding them that other people have much worse problems and still choose to live.	12.5	62.5	**75.0**
The first aider should tell the person to cheer up, and promise that everything will be okay.	4.2	62.5	**66.7**
If the suicidal person agrees to hand over the means of suicide, on the condition that they can have them back if they want them, the first aider should argue the point with them for as long as it takes.	4.2	66.7	**70.9**
If the intended means for suicide are of a less lethal type, the first aider doesn't need to worry about disabling the plan.	0	66.7	**66.7**
Contracts should include an agreement that the suicidal person stop thinking about suicide.	20.8	45.8	**66.6**
Contracts should be for a length of time that will present a challenge for the suicidal person, so that they can see that they can manage alone.	0	54.2	**54.2**
The first aider shouldn't use a contract with a suicidal person they know well.	8.3	45.8	**54.1**
The first aider shouldn't use a contract with a suicidal person who is severely depressed.	4.2	45.8	**50.0**
If a suicidal person asks the first aider to promise they will keep the discussion about suicide a secret, the first aider should keep the secret.	4.2	79.2	**83.4**
If a suicidal person asks the first aider to promise they will keep the discussion about suicide a secret, the helper should agree, but tell someone else anyway.	4.2	75	**79.2**
The first aider should encourage the suicidal person to take some sleeping pills, as they should be feeling better by the time they wake up.	0	100	**100.0**
The first aider should encourage the suicidal person to drink a few glasses of alcohol, to make the time pass more quickly.	4.2	95.8	**100.0**
Deliberate self-injury Round 1			
If the first aider interrupts someone who is deliberately injuring themselves - the first aider should leave immediately.	12.5	79.2	**91.7**
The first aider should ignore the injuries unless the person wants to talk about it.	12.5	45.8	**58.3**
The first aider should firmly say that self-injury is not acceptable behaviour.	8.3	45.8	**54.1**
If the first aider lives with the person, they should covertly ensure that sterile instruments are available for use (for example, clean blades in a bathroom cabinet).	8.3	41.7	**50.0**
The first aider should encourage the person to carry a first aid kit in order to minimise secondary harms from infection or poor healing.	8.3	45.8	**54.1**
The first aider should teach the person where they should not cut themselves, for example, the locations of major arteries which should be avoided.	4.2	54.2	**58.4**

The first aider should only seek professional mental health care if the person has agreed to it.	12.5	50	**62.5**
The first aider should be accepting of the person's right to injure themselves.	4.2	66.7	**70.9**
If the person is dissociating (seems spaced out, unaware of surroundings, or unresponsive) the first aider should not try to snap them out of it.	4.2	50	**54.2**
Deliberate self-injury Round 2			
The first aider should only stay with the person until help arrives only if the injury is serious.	10.5	63.2	**73.7**
